# Moderate Nrf2 Activation by Genetic Disruption of Keap1 Has Sex-Specific Effects on Bone Mass in Mice

**DOI:** 10.1038/s41598-019-57185-1

**Published:** 2020-01-15

**Authors:** Yukun Yin, Kylie A. Corry, John P. Loughran, Jiliang Li

**Affiliations:** 1 Department of Biology, Indiana University Purdue University Indianapolis, Indianapolis, IN 46202 USA; 2 Department of Traditional Chinese Medicine, National Cancer Center/National Clinical Research Center for Cancer/Cancer Hospital, Chinese Academy of Medical Sciences and Peking Union Medical College, Beijing, 100021 P.R. China

**Keywords:** Stress signalling, Bone

## Abstract

Keap1 is a negative controller of the transcription factor Nrf2 for its activity. The Keap1/Nrf2 signaling pathway has been considered as a master regulator of cytoprotective genes, and exists in many cell types including osteoblasts and osteoclasts. Our previous study shows Nrf2 deletion decreases bone formation. Recent studies show hyperactivation of Nrf2 causes osteopenia in Keap1^−/−^ mice, and Keap1^−/−^ osteoblasts have significantly less proliferative potential than Keap1^+/−^ osteoblasts. We aimed to examine if moderate Nrf2 activation by disruption of Keap1 impacts bone metabolism. We examined bone phenotype of Keap1 heterozygotic mice (Ht) in comparison with Keap1 wild type (WT) mice. Deletion or knockdown of Keap1 enhanced the gene expression of Nrf2, ALP and wnt5a in cultured primary osteoblasts compared to WT control. In male mice, compared with their age-matched littermate WT controls, Keap1 Ht mice showed significant increase in bone formation rate (+30.7%, *P* = 0.0029), but did not change the ultimate force (*P* < 0.01). The osteoclast cell numbers (−32.45%, P = 0.01) and surface (−32.58%, P = 0.03) were significantly reduced by Keap1 deficiency in male mice. Compared to male WT mice, serum bone resorption marker in male Keap1 Ht mice was significantly decreased. Our data suggest that moderate Nrf2 activation by disruption of Keap1 improved bone mass by regulating bone remodeling in male mice.

## Introduction

Our recent study has demonstrated that antioxidant factors may play a positive role in bone formation^1^. However, it is not clear if and how antioxidants affect skeletal development and bone homeostasis. The Kelch-like ECH-associated protein 1 (Keap1)/Nuclear factor erythroid 2-related factor 2 (Nrf2) protein axis plays an important role in regulating cytoprotective enzymes^2^. Keap1 negatively regulates Nrf2-dependent transcription of cytoprotective enzymes by inhibiting nuclear translocation of Nrf2, with cytoplasmic ubiquitination and degradation of Nrf2^3^. Nrf2 is sequestered in the cytoplasm until is activated, then acts as a regulator of cytoprotective genes against oxidative and chemical insults^4^. Nrf2 transcriptionally controls the gene expression of many cytoprotective enzymes, such as heme oxygenase-1 (HO-1)^2^, NAD-(P)H:quinone reductase (NQO1)^5^, γ-glutamylcysteine synthetase (GCS)^6^, and glucose-6-phosphate dehydrogenase^7^.

It is reported that reactive oxygen species (ROS) acts as intracellular signaling molecules to regulate osteoclast differentiation, and the Keap1/Nrf2 axis plays a role in osteoclast differentiation by regulating intracellular ROS signaling^8^. More studies revealed that deletion of Nrf2 reduces skeletal mechanical properties^1,9^, decreases load-driven bone formation^1^, impairs fracture healing in mice^10^, induces oxidative stress and promotes RANKL-induced osteoclast differentiation^11^. So Nrf2 is considered as playing an important role in the regulation of bone homeostasis in bone cells^12^. Recently, two Japanese labs have examined the role of Keap1 in skeletal development and bone homeostasis^13,14^. Because Keap1^−/−^ mice die within 3 weeks of birth due to severe hyperkeratosis in esophagus and forestomach^15^, one lab examined the newborn Keap1^−/−^ mice and found that talus and calcaneus bone formation is partially retarded^13^. Because the juvenile death of Keap1^−/−^ mice is caused by the obstruction of the esophagus and starvation^15^, the other lab generated a viable model by crossing the floxed Nrf2 mice, keratin5-drived-Cre mice and Keap1^−/−^ mice in which a squamous epithelium-specific Nrf2 deficiency in the context of systemic Keap1 deletion^14,16^. These unique mice (Keap1^−/−^; Nrf2^Flox/Flox^; Keratin5-Cre), which display a complication of nephrogenic diabetes insipidus^16^, present smaller bone size and lower bone density compared to the control mice despite the osteoclast number and bone resorption are significantly inhibited^14^. These data suggest that hyperactivation of Nrf2 markedly inhibits bone formation in Keap1^−/−^ mice. However, this paper has also demonstrated that Keap1^+/−^ osteoblasts have more significantly differentiative potential than Keap1^−/−^ osteoblasts by alkaline phosphatase staining^14^. So, we decided to examine if moderate Nrf2 activation in Keap1^+/−^ mice stimulates bone formation.

In this study, we hypothesized that moderate Nrf2 activation improves bone mass and strength in Keap1^+/−^ mice. To investigate this hypothesis, an *in vivo* mouse model and an *in vitro* cell culture system from Keap1 knockdown and knockout mice were utilized.

## Materials and Methods

### Experimental animals

All procedures performed in this study were in accordance with the Indiana University Animal Care and Use Committee Guidelines and were approved by Indiana University–Purdue University Indianapolis School of Science Animal Care and Use Committee.

Keap1^+/−^ mice with a mixed genetic background of C57BL6/129P2 were purchased from RIKEN BioResource Center of Japan (RBRC01388). Since mice without the Keap1 gene (Keap1^−/−^ mice) die before weaning due to the hyperkeratotic lesions in the esophagus and stomach, which led to obstruction of the upper digestive tract^15^, Keap1^+/−^ mice were used for these animal experiments. Male Keap1^+/+^ mice (WT, n = 11), male Keap1^+/−^ mice (Ht, n = 12), female Keap1^+/+^ mice (WT, n = 14), and female Keap1^+/−^ mice (Ht, n = 13) were used for the study. The mice were housed in plastic cages at 22 ± 1 °C on a 12-h light/12-h dark cycle with lights on from 6:00 AM to 6:00 PM. Standard rodent chow and water were provided ad libitum throughout the entire feeding period. Intraperitoneal injections of calcein (30 mg/kg body weight; Sigma) and alizarin (50 mg/kg body weight; Sigma) were administered 10 and 4 days before mice were sacrificed at the age of 18 weeks old. The left femurs of both genotypes were used to determine bone size, bone structure, and mechanical properties. The right distal femurs were sectioned and used for measurement of bone formation and resorption, and the midshaft parts were sectioned for the bone formation observation.

### Genotyping

**Figure 1:**
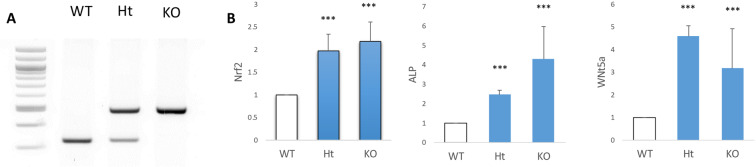
Genotypes and gene expression of Keap1^+/+^ (WT), Keap1^+/−^ (Ht) and Keap1^−/−^ (KO) mice and calvarial osteoblasts. (**A**) Gel electrophoresis of genotypes of Keap1 WT, Ht and KO mice. (**B**) The mRNA levels of Nrf2, ALP and wnt5a in Keap1 WT, Ht and KO calvarial osteoblasts were analyzed using real-time PCR (n = 3). Data were analyzed using one-way ANOVA followed by t test. Note that: ***P < 0.001 for comparison to WT osteoblasts.

To genotype the Keap1^+/+^, Keap1^+/−^ and Keap1^+/+^ mice, 1 mm of mouse tail was cut and put in tail lysis buffer (50 Mm Tris PH8.0 + 50 mM KCl + 2.5 mM EDTA + 4% NP-40 + 0.045% Tween-20) with 2% proteinase K in a 56°C water bath overnight and then 95°C dry bath for 10 minutes before 100 mL water was added. REDTaq ReadyMix ^TM^ PCR Reaction Mix was used for PCR. PCR conditions were: denaturing at 94 °C for 30 s, annealing at 55 °C for 30 s and extension at 68 °C for 30 s. PCR was conducted over 40 cycles and consisted of an initial denaturing step (94 °C, 1 min) and a final extension step (68 °C, 5 min) before a final hold at 4 °C. PCR products were subsequently separated on an agarose gel with SYBR DNA stain and photographed. Primer sequences for genotyping: Keap1-d132 (5′-CGGGATCCCCATGGAAAGGCTTATTG-3′), Keap1-TVneo (5′-TCAGAGCAGCCGATTGTCTGTTGTGCC-3′) and Keap1-intR (5′-CAGTTTTCCTCCAGCCTGTC-3′) were used. The WT showed one label around 350 bp, the KO was around 500 bp, and the Ht showed double labels in both 350 and 500 bp (Fig. 1A).

### Histomorphometry

Histomorphometry was carried out as described previously^1,17–19^. Bone specimens from mice were immersed in 10% neutral buffered formalin for 48 h. The specimens were then dehydrated in graded alcohols and methyl methacrylate, then embedded in methyl methacrylate with 4% of dibutyl phthalate and 0.25~0.8% of Perkadox × 16^3^. Using a diamond-embedded wire saw (Histo-saw; Delaware Diamond Knives, Wilmington, DE), transverse thick sections (50 μm) were cut at the ulna and femur midshafts, ground to a final thickness of 20~30 μm, and mounted on microscope slides. Three sections per limb were used for bone histomorphometry with a Nikon Optiphot fluorescence microscope (Nikon, Inc., Garden City, NJ) using a Bioquant digitizing system (R&M Biometrics, Nashville, TN). The following primary data were collected from the periosteal surface at 250 × magnification: total perimeter (B.Pm); single label perimeter (sL.Pm); double label perimeter (dL.Pm); and double label area (dL.Ar). From these primary data, the following quantities were derived: mineralizing surface (MS/BS = [1/2sL.Pm + dL.Pm]/B.Pm × 100; %); mineral apposition rate (MAR = dL.Ar/dL.Pm/6 days; μm/day); and bone formation rate (BFR/BS = MAR × MS/BS × 3.65; μm^3^/μm^2^ per year).

From the distal femurs, 5-μm-thick frontal sections were cut using a microtome (Leica, Germany). For each set of the bone sections from femurs, two unstained sections were mounted on microscope slides, while other sections were stained with tartrate-resistant acid phosphatase (TRAP) to identify active osteoclasts. The following primary data were collected from the metaphyseal area, 0.5 mm distal to the growth plate and 0.5 mm away from the intracortical surface, at 250 × magnification: tissue area (T.Ar), trabecular bone area (tB.Ar), trabecular bone perimeter (tB.Pm), single label perimeter (sL.Pm), double label perimeter (dL.Pm), double label area (dL.Ar), osteoclast surface (Oc.S), and osteoclast number (Oc.N). From these primary data, the following quantities were derived: bone volume (BV/TV = tB.Ar/T.Ar × 100; %), mineralizing surface (MS/BS = [1/2sL.Pm + dL.Pm]/B.Pm × 100; %), mineral apposition rate (MAR = dL.Ar/dL.Pm/6 days; μm/day), bone formation rate (BFR/BS = MAR × MS/BS × 3.65; μm^3^/μm^2^ per year), percentage of osteoclast surface (Oc.S/BS = Oc.S/B.Pm; %), and osteoclast number per mm (Oc.N/BS = Oc.N/B.Pm; #/mm).

### Micro computed tomography (μCT) analysis

The μCT scanning and analysis were carried out as described previously^1,17–19^. Left femurs were scanned using micro-CT (μCT) (SkyScan 1172, Bruker-microCT, Kontich, Belgium), followed by reconstruction for conversion into individual cross-section slices using the SkyScan NRecon software (Bruker-microCT, Kontich, Belgium). Bone X-ray profile images were then reconstructed into three-dimensional (3D) structure and ready for SkyScan CT-Analyser (CTAn) (Bruker-microCT, Kontich, Belgium) software analysis. One millimeter of trabecular bone located 1 mm above the growth plat was analyzed. In the binary images processing, the Index axis of the Histogram is set to 50 (the selected threshold). From the 3D analysis basic values and results, Bone Volume/Tissue Volume (BV/TV), Trabecular Bone Thickness (Tb.Th), Trabecular Bone Number (Tb.N), and Trabecular Bone Separation (Tb.Sp) were reported.

To evaluate the impact on cortical bone by Keap1 knockdown, mid-shaft femur’s cross-sectional geometric properties were analyzed. The femur mid-shaft position was determined by calculated half-length site of femur proximal end to distal end distance. Bone geometric properties were then analyzed from 7 mid-shaft sections (3 sections before + 1 midshaft section + 3 sections after) of each bone by using the SkyScan Software CTAn and a MATLAB script wrote by Professor Joseph Wallace at Indiana University-Purdue University Indianapolis, Department of Biomedical Engineering. For the measurement of geometric properties, each section was thresholded into bone and non-bone voxels using a previously defined method^19^. Mid-shaft femur geometric properties were reported as described before: cross-sectional area, cortical area, marrow area, average cortical thickness, anterior-posterior (AP) width, medial-lateral (ML) width, AP to ML Ratio, cortical area to total area percentage, and periosteal bone perimeter.

### Mechanical testing

The mechanical testing was carried out as described previously^1,17–19^. The left femurs obtained from 18-week-old mice were tested via three-point bending and compression, respectively, following previously published protocols^20^. Briefly, bones were brought to room temperature slowly (~2 h) in a saline bath, and loaded to failure at 2 mm/min with force versus displacement data collected at 10 Hz using a servo-hydraulic test system (Test Resources). Femurs were loaded to failure in an anterior–posterior direction with the upper contact area at the mid-diaphysis (50% total bone length), and the two bottom contact points positioned around this point, separated by 8 mm. Structural mechanical properties, determined ultimate force (FU; N), stiffness (S; N/mm), and work to failure (U; mJ) were determined from the load deformation curves using standard definitions. Note that FU represents the strength of the bone, whereas U is a measure of the energy required to break the bone.

### Primary cell culture

Primary osteoblasts were isolated from calvarial bone of 1~2-day-old Keap1^+/−^ (Ht) mice, Keap1^−/−^ (KO) mice and littermate controls by sequential digestions with collagenase and trypsin^20^. Calvarial cells were cultured and subcultured in α-Minimal Essential Medium (α-MEM; Sigma, St. Louis, MO, USA) containing 10% fetal bovine serum (FBS; Atlanta Biologicals, Norcross, GA), 100 U/ml penicillin G (Sigma) and 100 μg/ml streptomycin (Sigma). Cells were maintained in a 95% air/5% CO_2_ humidified incubator at 37 °C and subcultured every 72 hours.

### RNA isolation, reverse transcription and real-time PCR

Total RNA of the cultured calvarial osteoblasts was extracted using TRIzol reagent (Invitrogen). Using approximately 50 ng of total RNA, reverse transcription was conducted with a SuperScript III First-Strand Synthesis system kit (Invitrogen). Quantitative real-time PCR was performed using a FastStart Universal SYBR green PCR master kit (Roche). PCR primers were Nrf2 (forward 5′-TCTCCTCGCTGGAAAAAGAA-3′; reverse 5′-AATGTGCTGGCTGTGCTTTA-3′), alkaline phosphatase (ALP, forward 5′-GCTGATCATTCCCACGTTTT-3′; reverse 5′-CTGGGCCTGGTAGTTGTTGT-3′), Wnt5a (forward 5′-GTGGTCGCTAGGTATGAATAA-3′; reverse 5′-CGCGTATGTGAAGGCCGTC-3′) and GAPDH (forward 5′-ACTCCACTCACGGCAAATTC-3′; reverse 5′-TCTCCATGGTGGTGAAGACA-3′)^21^, where GAPDH was employed for internal control. The PCR results were interpreted using a delta CT method.

### Skeleton staining for newborn male mice

New born mice were anesthetized and removed of the skin and viscera. The forelimb and hindlimb of one side were taken for genotyping. The mice were fixed in 95% ethanol before being soaked in acetone to remove lipids. Then, the mice were moved into the staining solution (100% ethanol 80 mL + 0.3% Alcian Blue 8GX (dissolved in 70% ethanol) 10 mL + 0.3% Alizalin Red (dissolved in dH_2_O) 2 mL + Acetic Acid 10 mL) for 3 days. After a wash with 95% ethanol, the mice were soaked in 1% KOH once every day until the muscle was cleaned. The mice bones were finally stored in 8 parts of Glycerol and 2 parts of 1% KOH.

### Serum C-terminal telopeptides (CTX) of type I collagen

Serum concentration of the resorption marker CTX was measured using a commercially available ELISA kit (RatLaps, IDS Inc.). Blood samples were collected from the retromandibular vein, permitted to clot at room temperature for 30 minutes, spun at 13,000 rpm to separate the serum, and frozen at −80 °C. The thawed serum samples from the 18-week old mice of both genotype groups were assayed for CTX in triplicate according to the manufacturer’s instructions.

### Statistical analysis

Statistical analyses were computed with JMP (version 14, SAS Institute Inc.). Statistical details of each experiment with animal number can be found in the figure legends. Statistical significance was taken at p < 0.05. Data are presented as mean±SEM.

## Results

### The expression of Nrf2 in Keap1^+/−^ and Keap1^−/−^ osteoblasts

We examined Nrf2, ALP and WNT5a mRNA using primary calvarial osteoblasts of Keap1 WT, Ht and KO mice (Fig. 1B). The real-time PCR analysis showed that the Nrf2 mRNA level in Keap1 Ht and KO calvarial osteoblasts was significantly greater than Keap1 WT osteoblasts. Both ALP and wnt5a mRNA expression was also significantly greater in Keap1 Ht and KO osteoblasts than in the wild type controls.

### Deficiency of Keap1 increases bone mass in Keap1^+/−^ mice

**Figure 2:**
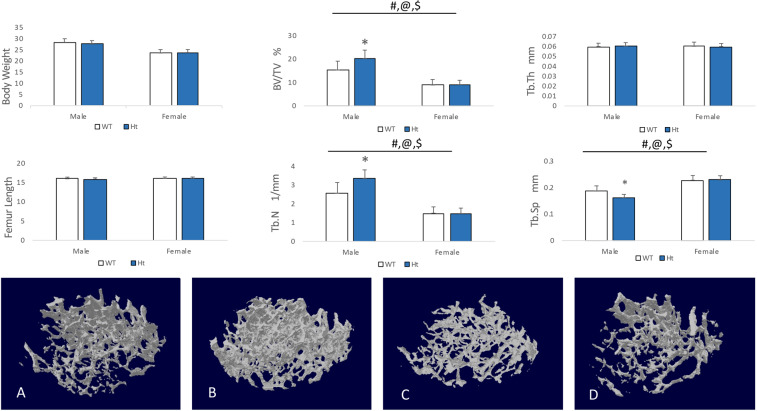
Bone volume and structure in Keap1 WT and Ht mice. There was no difference in body weight or femur length between WT and Ht in either male or female mice. Reconstruction of micro-CT images of distal femurs trabecular bone in (**A**) male wild type (WT), (**B**) male Keap1 heterozygous (Ht), (**C**) female wild type (WT) and (**D**) female Keap1 heterozygous (Ht) mice. Bone volume/tissue volume (BV/TV), thickness (Tb.Th), trabecular number (Tb.N) and trabecular separation (Tb.Sp) were determined. Results shown that BV/TV, Tb.N and Tb.Sp of male Keap1-Ht mice were significantly different compared to male WT mice. Data were tested using two-way ANOVA with Keap1 genotype and gender as main effects. The bar at the top of each graph indicates significance of the main effects and interaction (# = Keap1 genotype p < 0.05; @ = gender p < 0.05; $ = interaction p < 0.05). When at least one term was significant, t tests were conducted and are indicated as *p < 0.01 v.s. male WT. n = 10–14/group.

Knockdown of Keap1 did not affect the mouse femur length (WT vs. Ht: male: 15.96 ± 0.45 vs. 15.91 ± 0.33, *P* = 0.7449; female: 16.09 ± 0.35 vs. 16.11 ± 0.32, *P* = 0.8459) or body weight (WT vs. Ht: male: 28.37 ± 1.61 vs. 27.58 ± 1.54, *P* = 0.2472; female: 23.55 ± 1.55 vs. 23.76 ± 1.38, *P* = 0.7211, Fig. 2).

**Figure 3:**
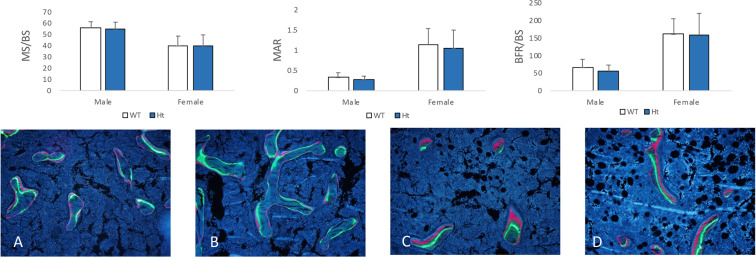
Trabecular bone in the distal femur with fluorescent labels (calcein and alizarin) in (**A**) male wild type (WT), (**B**) male Keap1 heterozygous (Ht), (**C**) female wild type (WT) and (**D**) female Keap1 heterozygous (Ht) mice, indicating mineralizing surface (MS/BS), mineral appositional rate (MAR) and bone formation rate (BFR/BS). There was no difference in MS/BS, MAR and BFR between WT and Ht mice in trabecular bone. Data were tested using two-way ANOVA with Keap1 genotype and gender as main effects.

The μCT analysis showed that, in male Keap1 Ht mice, the trabecular bone volume of distal femurs was significantly increased compared with their WT controls (BV/TV, +31.72%, *P* = 0.008, Fig. 2). Although no change was observed in the trabecular thickness (Tb.Th, +0.77%, *P* = 0.7707), there was significant difference found in trabecular number (Tb.N, +30.7%, *P* = 0.003) and trabecular separation (Tb.Sp, −13.21%, *P* = 0.0027) when compared to littermate controls. But there was no statistical difference in BV/TV between the female Keap1 Ht and WT mice. Bone formation parameters (MS/BS, MAR and BFR/BS) for the trabecular bone of distal femurs of Keap1 Ht mice showed no difference in comparison with WT control mice in either male or female (*P* > 0.05, Fig. 3).

**Figure 4:**
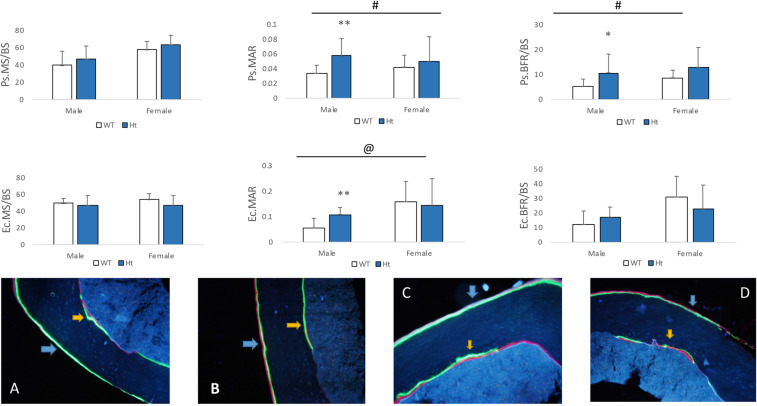
Cortical bone in the midshaft femur with fluorescent labels (calcein and alizarin) in (**A**) male wild type (WT), (**B**) male Keap1 heterozygous (Ht), (**C**) female wild type (WT) and (**D**) female Keap1 heterozygous (Ht) mice, indicating mineralizing surface (MS/BS), mineral appositional rate (MAR) and bone formation rate (BFR/BS). The cortical bone formation of male Keap1-Ht mice was increased significantly. Data were tested using two-way ANOVA with Keap1 genotype and gender as main effects. The bar at the top of each graph indicates significance of the main effects and interaction (# = Keap1 genotype p < 0.05; @ = gender p < 0.05; $ = interaction p < 0.05). When at least one term was significant, t tests were conducted and are indicated as **p < 0.05 and **p < 0.01 v.s. male WT mice. n = 9–14/group. Note that blue arrow: periosteal surface (Ps); yellow arrow: endocortical surface (Ec).

Analysis of cortical bone at the femoral mid-shafts revealed that MAR and BFR at the periosteal surface (Ps.MAR, +68.64%, *P* = 0.0084; Ps.BFR/BS, +107.87%, P = 0.0358) in male Keap1^+/−^ mice were significantly greater than the male controls (Fig. 4). At the endocortical surface, bone formation rate was not significantly different between male Keap1 ^+/−^ and WT mice. No significant difference in bone formation rate was found between female Keap1 Ht and WT mice.

### Deficiency of Keap1 reduces osteoclast number and activity in trabecular bone in Keap1^+/−^ mice

**Figure 5:**
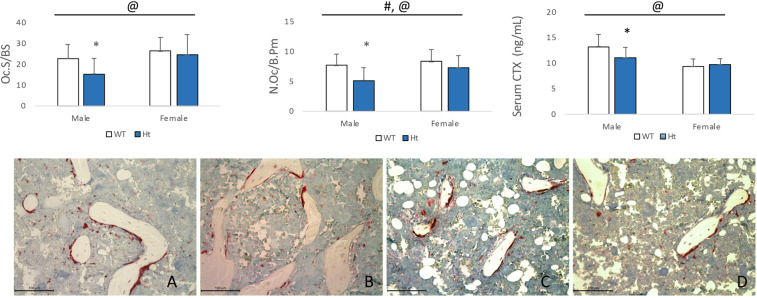
Trabecular bone in the distal femur stained by TRAP in (**A**) male wild type (WT), (**B**) male Keap1 heterozygous (Ht), (**C**) female wild type (WT) and (**D**) female Keap1 heterozygous (Ht) mice, indicating osteoclast surface (Oc.S/BS) and osteoclast number (N.Oc/B.Pm). The osteoclast number and surface were significantly reduced in male Keap1-Ht mice compared to the WT mice. Serum CTX was significantly less in male Keap1 Ht mice than male WT mice. Data were tested using two-way ANOVA with Keap1 genotype and gender as main effects. The bar at the top of each graph indicates significance of the main effects and interaction (# = Keap1 genotype p < 0.05; @ = gender p < 0.05; $ = interaction p < 0.05). When at least one term was significant, t tests were conducted and is indicated as *p < 0.05 v.s. male WT mice. For Oc.S/BS and N.Oc/B.Pm, n = 10–14/group. For CTX, n = 10–13/group.

The trabecular bone of the distal femur was stained by tartrate-resistant acid phosphatase (TRAP). The osteoclast cells were stained into a purple red color. Osteoclast cell number on bone surface (N.Oc/B.Pm) and osteoclast cell surface (Oc.S/BS) was measured. In the male Keap1 Ht mice, the N.Oc/B.Pm (−32.45%, *P* = 0.0111) and Oc.S/BS (−32.58%, *P* = 0.0263) were both reduced significantly compared with their WT controls (Fig. 5). There was no difference in osteoclast number between female Keap1 Ht and WT groups.

### The bone resorption marker, CTX, was decreased in Keap1^+/−^ mice

The serum CTX in male Keap1+/− mice was significantly less (−16.60%, *P* = 0.0265) compared with their littermate controls, suggesting decreased bone resorption in male Keap1+/− mice. There was no statistical difference in serum CTX between female HT and WT mice (Fig. 5).

### Lack of Keap1 did not affect biomechanical properties of femurs

**Figure 6:**
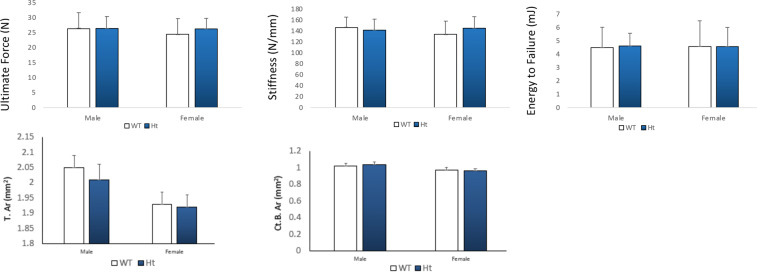
Mechanical properties and femoral cortical bone geometry were analyzed. In order to evaluate the mechanical properties of bone in Keap1^+/−^ mice, the femurs were subjected to three points bending test. There were no differences in the ultimate force, stiffness and energy to failure between Keap1 WT and Ht mice. There were not difference between total cross sectional area (T.Ar) and cortical bone area (Ct.B.Ar) between WT and Keap1^+/−^ mice in either male and female mice. Data were tested using two-way ANOVA with Keap1 genotype and gender as main effects. n = 11–14/group.

The mechanical properties of femurs in Keap1^+/−^ were evaluated by a three points bending test. At femoral midshafts, the ultimate forces, energy to failure and stiffness were reported. The results reveal that a lack of Keap1 did not change the bone strength of femurs in both male or female mice (Fig. 6).

### Newborn Keap1 Knockdown and knockout mice showed rapid bone mineralization

**Figure 7:**
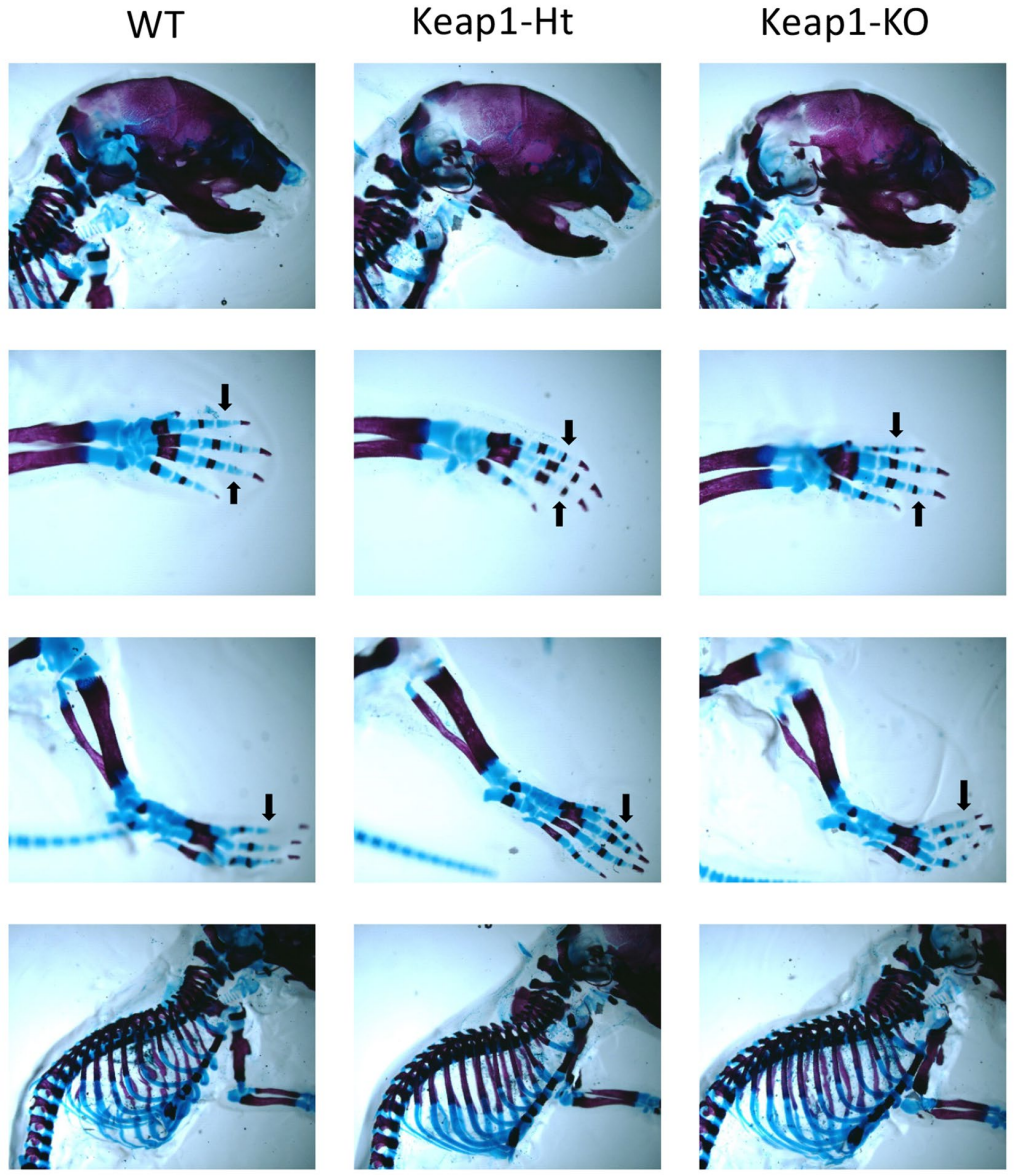
Keap1 deficient mice exhibit increased bone mineralization in male Keap1^+/−^ newborn mice compared to WT newborn mice. Newborn pups were stained with Alcian blue/Alizarin red. Bone is stained red and cartilage blue. Neurocranium, limbs and spine were detected. Limbs showed accelerated skeletal mineralization in fingers and toes (black arrows) in Keap1 heterozygotes and knockout newborn mice in comparison with their wild type control.

Only newborn male mice were stained to examine the skeletal development. The cartilage was stained in a blue color, while the bone was stained in purple. The Keap1 Ht and Keap1 KO mice showed more bone mineralization in their limbs compared with their littermate controls (Fig. 7), suggesting Keap1 deficiency accelerate skeletal mineralization.

## Discussion

The current study demonstrated that a lack of Keap1 enhances Nrf2 expression and leads to a higher bone mass in male adult mice. Keap1 deficiency suppressed osteoclast number and activity at the trabecular bone area and increased bone formation at the midshaft of femurs. The results revealed that Keap1/Nrf2 signaling plays an important role in bone modeling and remodeling.

Nrf2 activation by disruption of Keap1 negatively regulates osteoclastogenesis and bone resorption. Consistent with the previous studies^8,13,14^, our current study showed Keap1 deficiency resulted in a reduction in an osteoclast cell number and surface at the distal femur, which suggests a reduced bone resorption in Keap1^+/−^ mice. The serum CTX level was also lower in Keap1 Ht mice than the wild type mice, further suggesting that a lack of Keap1 systemically reduced bone resorption. Nrf2 activation by disruption of Keap1 inhibits ROS accumulation and downregulates NRATc1 and subsequently decreases osteoclast differentiation and activity^13^. Our previous study demonstrates that Nrf2 deletion leads to a higher bone resorption as shown by both the increased osteoclast cell number and higher expression of RANKL^1^. All these data indicate that Keap1/Nrf2 signaling pathway regulates bone resorption.

Bone formation was increased by Keap1 knockdown in this study as evidenced by greater MAR and bone formation rate at the midshaft of femurs of Keap1 Ht mice in comparison with the WT controls. The greater MAR suggests more active osteoblasts in Keap1 Ht mice. The cell culture study using calvarial bone cells further showed higher ALP and Wnt5a mRNA in Keap1 Ht osteoblasts than the wild type osteoblasts, suggesting Keap1 deficiency stimulates osteoblast differentiation. These data are supported by our previous reported results showing Nrf2 deletion decreases bone formation^1^. Overall, these data suggest that Keap1/Nrf2 signaling plays an important role in osteoblast differentiation and activity.

Inhibition of bone resorption and increase in bone formation led to higher bone mass in male Keap1^+/−^ mice in comparison with WT controls in this study. These data are different from two previous reports showing the Keap1^−/−^ mice have defective mineralization and lower bone mass compared to the controls. Using the same whole skeleton staining, our data showed the accelerated bone mineralization in Keap1^−/−^ newborn pups, contrary to the previous study showing a defect in mineralization^13^. Further, another study using a complicated genetically modified mouse model (Keap1^−/−^; Nrf2^Flox/Flox^; Keratin5-Cre), as described previously, demonstrated that hyperactivation of Nrf2 leads to a smaller body size and lower bone density compared to the control mice (8–10 weeks male mice)^14^. Besides esophagus and stomach, Keratin 5 is expressed in thymus, uterus, prostate, mammary gland, etc^22^. Keratin 5 driven cre might cause Nrf2 deficiency in some endocrine glands which might indirectly affect bone metabolism. These unique mice also display a complication of nephrogenic diabetes insipidus^16^, which makes it difficult to explain the low bone mass phenotype. At the cellular level, our data show Keap1^−/−^ osteoblasts have similar or higher anabolism than Keap1^+/−^ osteoblasts as evidenced by qPCR data, whereas, the previous study shows that Keap1^−/−^ osteoblasts have significantly less differentiative potential than Keap1^+/−^ osteoblasts^14^. Although the effects of hyperactivation of Nrf2 in Keap1^−/−^ mice are still debatable, the moderate activation of Nrf2 enhances bone mass in in Keap1^+/−^ mice. Osteoblast-specific Keap1 deficient mice may help to clarify the discrepancy in the role of Keap1/Nrf2 in bone formation.

Our previous research demonstrated that Nrf2 knockdown reduced the energy to failure, ultimate forces and stiffness of femurs and lumbar vertebral bodies^1^, which means Nrf2 may play a role in maintaining bone strength. However, in this current study, when Keap1 was knocked down, the bone strength of femurs was not increased compared with the wild type controls. These data suggest enhanced Nrf2 expression does not increase bone strength in adult mice. It remains to be clarified whether Keap1/Nrf2 signaling pathway may help to preserve bone strength in aged mice.

Previous studies with Nrf2 knockout (KO) mice have shown sexual dimorphism in the skeletal phenotype of Nrf2 KO mice^1,9,23,24^. Female Nrf2 KO mice always show lower bone mass than the female WT mice^9,24^. But, compared to the male WT mice, male Nrf2 KO mice show either higher or lower bone mass^1,23,24^. In our current study, Nrf2 activation by disruption of Keap1 also presents sexual dimorphism in bone phenotype of Keap1^+/−^ mice, higher bone mass in male Keap1^+/−^ mice, but not in female Keap1^+/−^ mice compared to their sex specific controls. Because either Nrf2 deficiency or activation affects bone remodeling, in general, these data suggest that Keap1/Nrf2 signaling plays a critical role in both male and female bone tissues. However, the crosstalk between Nrf2 activation and signaling downstream of the sex steroid receptors needs to be further investigated.

There are limitations in this study. Because the global Keap1^+/−^ mice were used, it is difficult to exclude the influence of endocrine systemic factors regulated by Keap1 on bone tissues. In order to study the specific role of Keap1 in different cell population, investigation of osteoclast- and osteoblast-specific Keap1 knockout mouse models would provide more insight into the role of Keap1/Nrf2 signaling in bone metabolism and mechanical properties. Further, since Keap1/Nrf2 signaling pathway activates most of cytoprotective genes, it would be more valuable to examine the role of Keap1/Nrf2 signaling in osteoclasts and osteoblasts in aging mice.

In conclusion, loss-of-function mutations of Keap1 increase bone mass, reduces bone resorption; Keap1 plays a critical role in the bone metabolism and its effects on bone are sex specific.

## Data Availability

The data that support the findings of this study are available from the corresponding authors upon reasonable request.
