# Author Correction: Moderate Nrf2 Activation by Genetic Disruption of Keap1 Has Sex-Specific Effects on Bone Mass in Mice

**DOI:** 10.1038/s41598-021-87424-3

**Published:** 2021-05-10

**Authors:** Yukun Yin, Kylie A. Corry, John P. Loughran, Jiliang Li

**Affiliations:** 1 Department of Biology, Indiana University Purdue University Indianapolis, Indianapolis, IN 46202 USA; 2 Department of Traditional Chinese Medicine, National Cancer Center/National Clinical Research Center for Cancer/Cancer Hospital, Chinese Academy of Medical Sciences and Peking Union Medical College, Beijing, 100021 P.R. China

Correction to: *Scientific Reports* https://doi.org/10.1038/s41598-019-57185-1, published online 15 January 2020

The original version of this Article contained an error in Affiliation 2, which was incorrectly given as ‘Department of Traditional Chinese Medicine, National Cancer Center/Cancer Hospital, Chinese Academy of Medical Sciences and Peking Union Medical College, Beijing, 100021, P.R. China′. The correct affiliation is listed below:

Department of Traditional Chinese Medicine, National Cancer Center/National Clinical Research Center for Cancer/Cancer Hospital, Chinese Academy of Medical Sciences and Peking Union Medical College, Beijing, 100021, P.R. China.

This error has now been corrected in the PDF and HTML versions of the Article.

